# Investigation of Correlations Between Optical Coherence Tomography Biomarkers and Visual Acuity in X-Linked Retinoschisis

**DOI:** 10.3389/fmed.2021.734888

**Published:** 2022-01-27

**Authors:** Zhanjie Lin, Siwen Zang, Dan Jouma Amadou Maman Lawali, Yu Xiao, Xiaomin Zeng, Honghua Yu, Yijun Hu

**Affiliations:** ^1^Department of Ophthalmology, Guangdong Eye Institute, Guangdong Provincial People's Hospital, Guangdong Academy of Medical Sciences, Guangzhou, China; ^2^Graduate School, Shantou University Medical College, Shantou, China; ^3^State Key Laboratory of Ophthalmology, Zhongshan Ophthalmic Center, Sun Yat-sen University, Guangzhou, China; ^4^Aier Institute of Refractive Surgery, Refractive Surgery Center, Guangzhou Aier Eye Hospital, Guangzhou, China; ^5^Aier School of Ophthalmology, Central South University, Changsha, China

**Keywords:** X-linked retinoschisis, optical coherence tomography, visual acuity, correlation, biomarker

## Abstract

**Purpose:**

To investigate the imaging biomarkers of spectral-domain optical coherence tomography (SD-OCT) and their correlations with age and best-corrected visual acuity (BCVA) in patients with X-linked retinoschisis (XLRS).

**Methods:**

OCT images of 72 eyes of 39 patients with confirmed XLRS were obtained to assess imaging biomarkers, including but not limited to the automatic evaluation of foveal thickness, central subfield thickness (CST), macular volume, and the manual measurement of area of macular schisis cavity (AMS). Correlations between age/BCVA and all OCT parameters were computed as well.

**Results:**

In this study, median age was 10.5 (8–12) years old and median BCVA was 0.90 (0.70–1.00) logarithm of the minimum angle of resolution. Macular retinoschisis was found in all affected eyes, with peripheral retinoschisis (PRS) in 34 (47.2%) eyes. Cystic cavities most frequently affected inner nuclear layer (100%) in the macula. Ellipsoid zone (EZ) defects occurred in 53 (73.6%) eyes. As for correlation, BCVA was significantly correlated with several OCT parameters, including CST, AMS, EZ defect, PRS and vitreomacular adhesion, whereas no correlation was found between age and any OCT parameter.

**Conclusion:**

Explicable OCT imaging biomarkers such as CST, AMS, and photoreceptor defects were identified and may serve as reference parameters or potential regions of interest for future observational and interventional research design and result interpretation.

## Introduction

X-linked retinoschisis (XLRS), an inherited retinal dystrophy caused by *RS1* gene mutations, is one of the leading causes of macular degeneration in juvenile males, with an estimated prevalence ranging from 1:30,000 to 1:15,000 (1, 2). Most disease-causing *RS1* gene mutations are identified to alter the formation or secretion of an adhesive protein named retinoschisin, resulting in weakened adhesion between each retinal layer and therefore the formation of schisis cavities within the retina (2, 3). Although visual acuity can be substantially compromised by complications such as vitreous hemorrhage (up to 1/3 of patients) and retinal detachment (up to 20% of patients) over the natural history of disease, most XLRS patients only present a mild or moderate visual impairment at an early age, widely varying from 20/20 to 20/600 (4, 5). Therefore, interpretations of the morphological changes and their relationship with visual function in XLRS patients are of great clinical significance. Previous imaging-based investigations have attempted not only to reveal the correlations between retinal structure and function, but also to qualitatively or quantitatively evaluate the structural and functional outcomes of various treatments; however, the built-in measurements of optical coherence tomography (OCT) devices are limited and the correlations found in prior studies showed insufficient consistency (6–11).

In this present study, we investigated the retinal morphological characteristics in XLRS patients by using spectral-domain OCT (SD-OCT). To help better understanding of visual impairment, we further evaluated the correlations between age/best-corrected visual acuity (BCVA) and structural defects to identify convincing imaging biomarkers in OCT. Such findings may provide solid and explicable reference parameters or potential regions of interest for future observational and interventional researches.

## Materials and Methods

Patients diagnosed with XLRS on SD-OCT and confirmed by the detection of *RS1* gene mutations in Zhongshan Ophthalmic Center, Sun Yat-sen University between 2011 and 2014 were evaluated. Exclusion criteria involved (1) poor OCT image quality insufficient to identify retinal structural defects, (2) patients with anterior segment abnormalities found in the slit-lamp examination, or other complications hindering ophthalmic observation, e.g., vitreous hemorrhage and retinal detachment, and (3) patients with history of vitreoretinal surgery or retinal laser photocoagulation. Eventually, a total of 39 male patients and 72 affected eyes were included in this retrospective, observational, single-center case series study. This study was conducted in adherence to the tenets of the Declaration of Helsinki and received approval from the Medical Ethics Committee of Zhongshan Ophthalmic Center, Sun Yat-sen University, Guangzhou, China. Informed consents were obtained from all patients or their legal guardians.

All the patients underwent a series of comprehensive ophthalmic examinations, including autorefraction, BCVA measured by a logarithm of the minimum angle of resolution (logMAR) chart, intraocular pressure, slit-lamp biomicroscopy, dilated funduscopic examination, and SD-OCT scanning. Genomic DNA was also extracted from peripheral blood leukocytes for gene sequencing to confirm genetic diagnosis.

**Figure 1:**
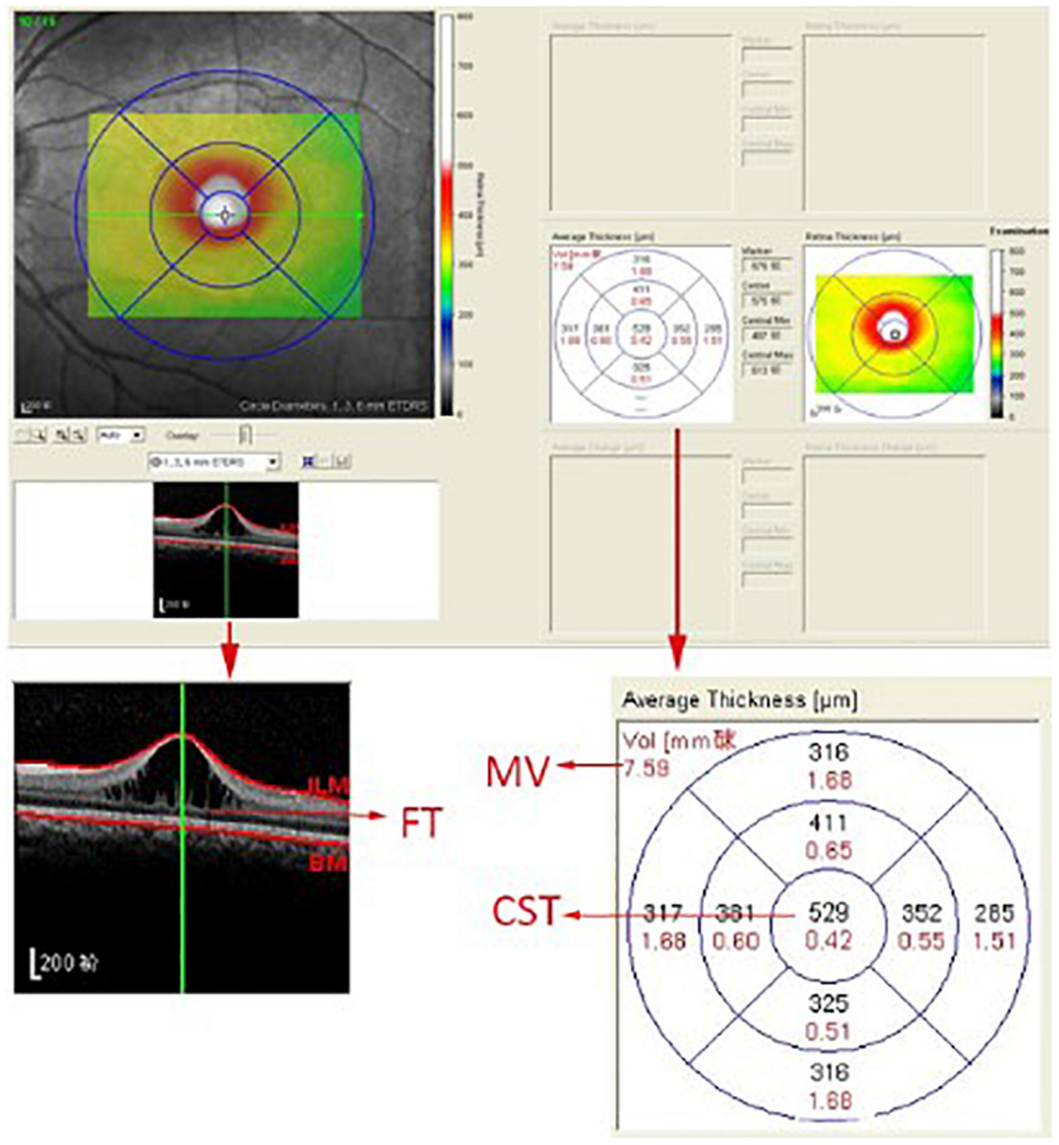
The computer user interface of the spectral-domain optical coherence tomography. The foveal thickness (FT), central subfield thickness (CST), and macular volume (MV) were automatically measured by the built-in software of the spectral-domain optical coherence tomography device.

**Figure 2:**
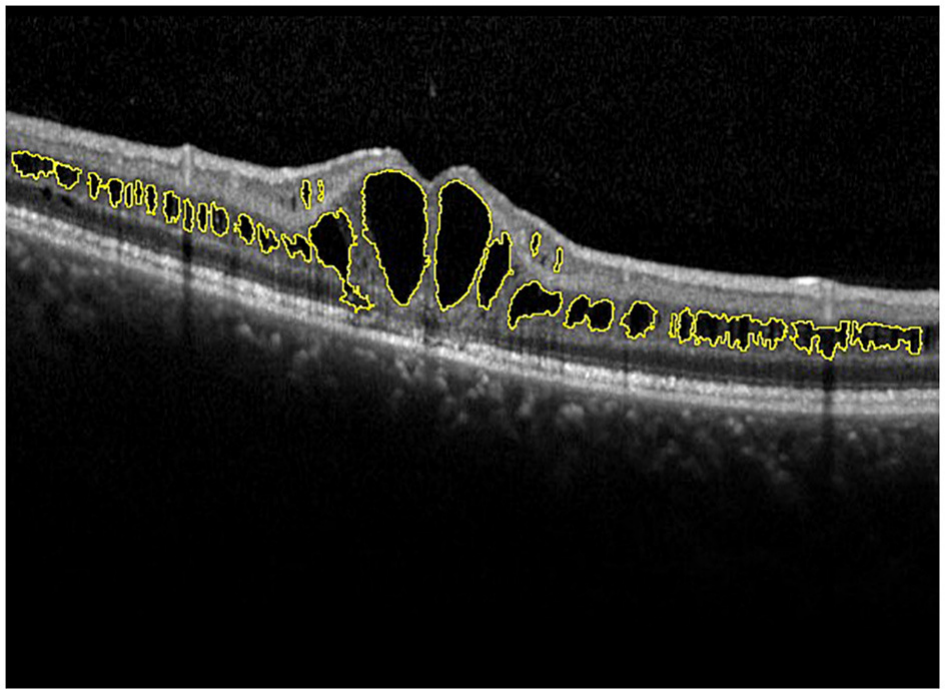
Manual measurement of the area of macular schisis cavity (AMS). The macular schisis cavities were marked by yellow lines and the results were computed by ImageJ software.

A skilled technician performed SD-OCT (Spectralis, Heidelberg Engineering, Heidelberg, Germany) examination to all XLRS patients. The scanning area was 6 × 6 mm centered at the fovea, generating a set of 25 horizontal and vertical cross-sectional B-scan images. The technician would manually adjust the center to the fovea only when the device had failed, based on the anatomical nature and hyporeflective appearance of the fovea. Images were evaluated by the built-in software of SD-OCT device to measure the foveal thickness (FT), central subfield thickness (CST), and macular volume (MV) (Figure 1). FT was defined as the mean center point thickness of the two horizontal and vertical scans through the fovea. CST was defined as the mean retinal thickness of the central 1-mm diameter circle of the Early Treatment Diabetic Retinopathy Study grid, whereas MV was calculated by the summation of all the volumes obtained in the nine subfields (12, 13). In addition, two retinal specialists independently measured the area of macular schisis cavity (AMS) in horizontal images, with the help of ImageJ software (U. S. National Institutes of Health, Bethesda, Maryland, USA, https://imagej.nih.gov/ij/, Figure 2). Specifically, they manually marked all the dark regions between internal limiting membrane and retinal pigment epithelium (RPE) in each scan, and then eliminated the non-cystic changes as well as those caused by artifacts before summation into total AMS. Each specialist measured twice and the mean value was used for statistical analysis. Ellipsoid zone (EZ) defect, macular retinoschisis (MRS), peripheral retinoschisis (PRS), vitreomacular adhesion (VMA), and macular atrophy (MA) were also independently assessed by the two specialists, using both horizontal and vertical SD-OCT scans. EZ defect was defined as the irregularity or disruption of the EZ layer that was not caused by shadowing throughout the macula, whereas MRS and PRS were designated as the presence of schisis cavity in their respective regions (7, 8). Besides, the definition of VMA was consistent with the anatomical criteria demonstrated by the International Vitreomacular Traction Study Group and the European Eye Epidemiology consortium, whereas the evidence of MA was supported by a hypopigmented lesion within the macula found in fundus photograph, usually characterized as a well-defined, grayish-white or whitish chorioretinal atrophic lesion (9, 14, 15). If there was a disagreement regarding the abovementioned qualitative descriptions, an experienced retinal professor would intervene and make the final judgement.

Statistical analysis was performed by SPSS version 20 (SPSS Inc., Chicago, Illinois, USA). All quantitative variables underwent the Shapiro-Wilk normality test. Data of normal distribution were presented as mean ± standard deviation, whereas those of non-normal distribution as median (interquartile range). Percentage was used to describe qualitative variables. Moreover, partial correlation was performed to evaluate correlations between age/BCVA and OCT parameters while adjusting for the intereye correlation, and corresponding correlation coefficients were designated as *r*_*p*_ and *r*_*s*_. A *p* < 0.05 was considered statistically significant.

## Results

A total of 72 eyes of 39 XLRS patients, with median age of 10.5 (8–12) years old, were included consecutively in this study, among which 33 (84.6%) patients were affected bilaterally. Overall, the patients presented with median BCVA of 0.90 (0.70–1.00) logMAR upon visual acuity examination. A representative fundus photograph is shown in Supplementary Figure 1.

**Figure 3:**
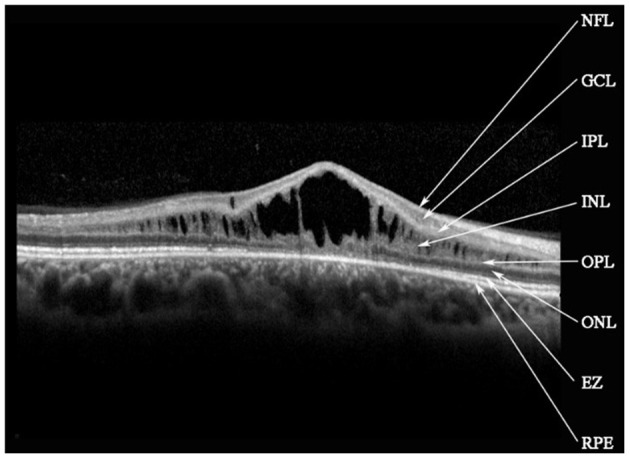
Spectral-domain optical coherence tomography image reveals macular schisis cavities in inner nuclear layer (INL), outer plexiform layer (OPL), and outer nuclear layer (ONL). Small cystic cavities were found in the ganglion cell layer (GCL). NFL, nerve fiber layer; IPL, inner plexiform layer; EZ, ellipsoid zone; RPE, retinal pigment epithelium.

**Figure 4:**
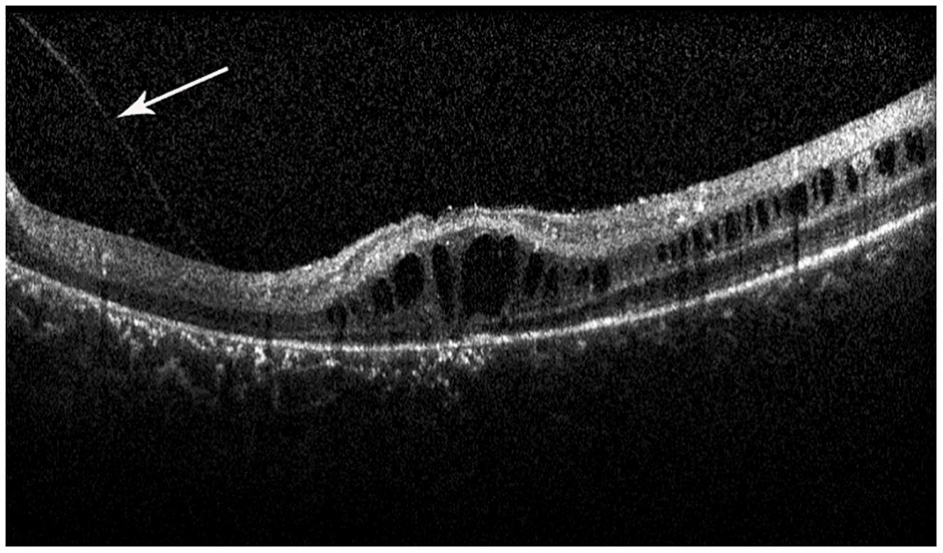
Spectral-domain optical coherence tomography image reveals the vitreomacular adhesion (VMA) in the macula (white arrow), with loss of outer retinal layers, and a minimal attenuation of RPE band. But the patient did not have macular atrophy visible in the fundus photograph.

Retinal schisis cavities were found in the macula of all (100%) affected eyes, characterized by the typical spoke-like schisis or cystic changes (Figure 3), as well as in the retinal periphery of 34 (47.2%) eyes. Macular schisis affected inner nuclear layer in all 72 (100%) eyes, outer plexiform layer in 32 (44.4%) eyes, inner plexiform layer in 28 (38.9%) eyes, and outer nuclear layer in 13 (18.1%) eyes, with small cystic changes present in ganglion cell layer in 12 (16.7%) eyes. Overall, six (8.3%) eyes had schisis in all the five retinal layers. However, contrary to the abundant findings on MRS and PRS in affected eyes, we observed no MA in this XLRS population. EZ layer was found irregular or disrupted in 53 (73.6%) eyes, whereas VMA was found in 8 eyes (11.1%, Figure 4). With regards to quantitative values, all the variables were non-normally distributed. FT was measured as 460.50 (369.75–670.75) μm and CST as 490.00 (391.00–598.00) μm. MV was computed as 9.00 (7.07–10.13) mm^3^, and AMS as 0.827 (0.433–1.293) mm^2^.

**Figure 5:**
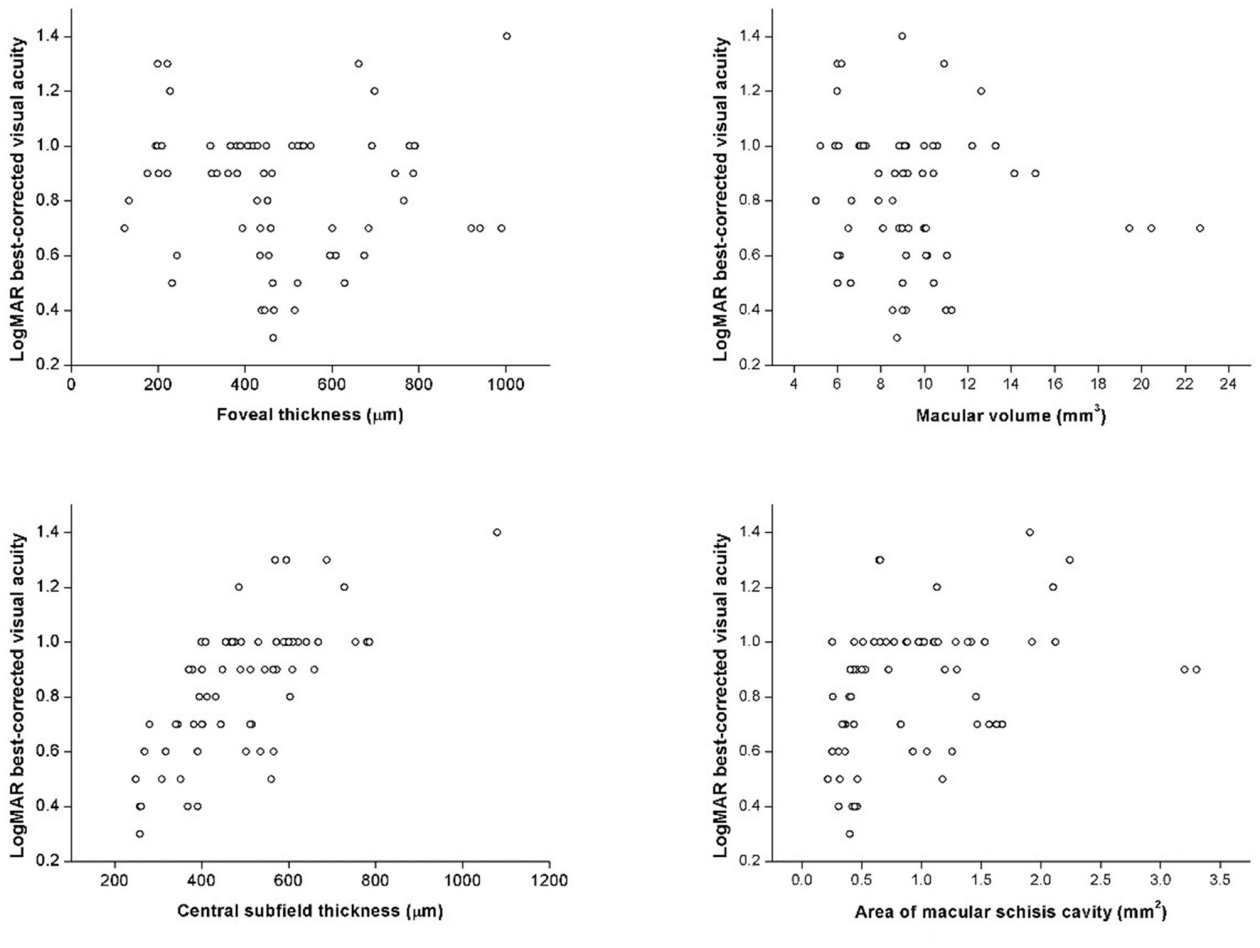
The scatter plots represent the linear correlations between BCVA and structural properties determined by spectral-domain optical coherence tomography.

In correlation analysis, no correlation was found between age and any OCT parameter. Contrarily, BCVA was significantly correlated with several OCT parameters, such as CST (*r*_*s*_ = 0.739, *p* < 0.001) and AMS (*r*_*s*_ = 0.451, *p* < 0.001), but not FT (*r*_*s*_ = −0.042, *p* = 0.094) or MV (*r*_*s*_ = −0.076, *p* = 0.532) (Figure 5). BCVA was also correlated with EZ defect (*r*_*s*_ = 0.585, *p* < 0.001), PRS (*r*_*s*_ = 0.515, *p* < 0.001), and VMA (*r*_*s*_ = 0.253, *p* = 0.035).

## Discussion

In this present study, we included 72 eyes from 39 XLRS patients for OCT examination and image quantification. Herein, the median age was 10.5 (8–12) years old with median BCVA of 0.90 (0.70–1.00) logMAR, and 33 (84.6%) patients had bilateral retinoschisis. MRS was found in all eyes with typical spoke-like cystic changes, whereas PRS was found in about half (47.2%) of the affected eyes. Besides, inner nuclear layer was the most affected retinal layer (100%) in MRS, followed by outer (44.4%) and inner (38.9%) plexiform layers. On the contrary, no MA was found in our study. EZ defect was identified in more than two thirds of the eyes (73.6%), with VMA identified in a much smaller amount (11.1%). For quantitative values, FT, CST, MV, and AMS were computed as 460.50 (369.75–670.75) μm, 490.00 (391.00–598.00) μm, 9.00 (7.07–10.13) mm^3^, and 0.827 (0.433–1.293) mm^2^, respectively. Correlation analysis revealed that BCVA was significantly correlated with several OCT parameters, including CST, AMS, EZ defect, PRS, and VMA.

Consistent with the general perception, findings of our study demonstrated that XLRS was an early-onset hereditary maculopathy in juvenile males, predominantly with bilateral and mild to moderate visual impairment (2, 4). We also found MRS in all eyes and PRS in 47.2% of them, which agreed with the former findings stating that schisis cavity usually presented in the macula of most, if not all, affected eyes, whereas peripheral retinoschisis roughly presented in half of the cases (3, 5, 8, 16, 17). Besides, similar to findings of previous investigations, such cystic changes were mostly confined within the inner retinal layers, with inner nuclear layer being the most commonly affected one in XLRS; however, there was discordance in the occurrence of defects in other layers (3, 6, 8, 18). Contrary to a previous study reporting MA occurrence of 11% in a 52-patient cohort, no (0%) macular atrophy was noted in our study (8). This difference could be explained by the fact that our patients of interest were relatively younger, aging 5–33 vs. 3–57 years old, since retinal atrophy has been widely demonstrated as an age-related, chronologically progressive manifestation in XLRS, which possibly resulted by the collapsed schisis cavities and RPE atrophy (8, 11, 19).

Nowadays, retinoschisin is commonly perceived as a cell-to-cell adhesive protein to maintain the integrity of the retina, especially the structure of photoreceptor-bipolar synapse. Thus, its depletion and dysfunction should disrupt the retinal photoreceptor microstructures, such as external limiting membrane and EZ (2, 3, 6, 20, 21). Although previous studies showed no specific order in the involvement or preference of these two layers, these defects were found correlated with visual acuity, even though some studies prior to the nomenclature system proposed by the International Nomenclature for Optical Coherence Tomography Panel addressed such defects in a different way (6, 8, 22). In our study, EZ defect presented in 73.6% of all eyes, and was associated with worse visual acuity, suggesting that photoreceptor defects should be responsible for the functional impairment in XLRS patients.

Correlations of age and OCT parameters were evaluated, but no significant result was found, which suggested that these OCT parameters might not be age-related. Such finding actually contradicted some of the results from previous studies, claiming that age was correlated with FT, CST, and AMS, although described in different terms (7, 8, 11). Nevertheless, this discordance could be caused by the differences of age range among study populations, with 7.8–79, 3–57, and 3–68 years of the abovementioned studies and 5–33 years in our study. As a result, our study might have failed to detect the possible tendency of underlying morphological changes in XLRS patients throughout the natural progression of the disease. Similarly, our patients of interest were relatively young so that BCVA would remain rather stable before the emergence of MA, which accounts for no significant correlation found between age and BCVA in our study as well.

Correlations between BCVA and quantitative OCT measurements showed interesting results: BCVA was correlated with CST and AMS but not FT or MV. In former studies, the role of CST remained rather ambiguous as some found it significantly associated with BCVA while others did not (6, 8, 9, 11). In our study, both thicknesses were evaluated, but only CST showed strong, significant positive correlation (*r*_*s*_ = 0.717, *p* < 0.001) with logMAR BCVA. CST, the average thickness within 1-mm diameter circle, contains more spatial information than the point-thickness measurement of FT. Therefore, it is not surprising that CST was inclined to better represent the morphological changes of the distorted macula. Mixed findings also appeared in prior studies with regard to MV and AMS; however in our study, significant correlation was found between BCVA and AMS instead of MV (7, 11, 19). It has been demonstrated that the inaccuracy of MV mainly came from the opposing effect of thickened inner retina and thinned outer retina, which are resulted by large, extensive schisis cavities and RPE atrophy, respectively (19). Such opposing effect caused biased MV measurement and thus inadequate explicability. In this sense, AMS was a better reference parameter as it exclusively regarded the area of schisis cavity as an essential retinal lesion. Therefore, significant correlation found between BCVA and AMS instead of MV provides a novel aspect in image evaluation and interpretation. Future investigations of morphological changes and their correlations with retinal function can rely on CST and AMS as descriptive variables. Hence, CST and AMS should be taken into consideration in subsequent researches on visual loss assessment, which provides a new perspective for discovering underlying physiological and pathological mechanism.

There are several limitations of the study. Firstly, the sample size is small with limited number of patients included in the study. Our findings may need to be validated in future studies with a larger sample size. Since most of the mutations in the present study were scattered in different locations of the *RS1* gene, the small sample size was not enough to perform a statistically powerful genotype–phenotype correlation. Secondly, no longitudinal follow-up was performed for the patients, and we could not observe the changes of OCT biomarkers of XLRS and their correlations with BCVA over time. Thirdly, we did not include functional studies such as microperimetry and electroretinography. It would be meaningful to investigate the correlations between OCT biomarkers and retinal functional metrics in patients with XLRS.

In conclusion, we investigated the retinal morphological characteristics and their correlations with age and BCVA in XLRS patients using SD-OCT. As a result, explicable imaging biomarkers such as CST, AMS, and photoreceptor defects were identified and may serve as reference parameters or potential regions of interest for future observational and interventional research design and result interpretation.

## Data Availability Statement

The raw data supporting the conclusions of this article will be made available by the authors, without undue reservation.

## Ethics Statement

The studies involving human participants were reviewed and approved by Medical Ethics Committee of Zhongshan Ophthalmic Center, Sun Yat-sen University, Guangzhou, China. Written informed consent to participate in this study was provided by the participants' legal guardian/next of kin.

## Author Contributions

HY and YH contributed to conception and design of the study, as well as data collection and management. YX and XZ performed the statistical analysis. ZL, SZ, and DM interpreted the results and co-wrote the manuscript. All authors contributed to manuscript revision and approved the submitted version.

## Funding

This work was supported by Grant 81870663 from the National Natural Science Foundation of China (HY), Grant KJ012019087 of the Outstanding Young Talent Trainee Program of Guangdong Provincial People's Hospital (HY), Grant KJ012019457 from the GDPH Scientific Research Funds for Leading Medical Talents and Distinguished Young Scholars in Guangdong Province (HY), Grant Y012018145 from the Talent Introduction Fund of Guangdong Provincial People's Hospital (HY), Grant A2021378 from the Medical Scientific Research Foundation of Guangdong Province, China (YH), Grant 2018SK50106 from the Technology Innovation Guidance Program of Hunan Province (YH), Grant AM1909D2 and AR1909D2 from the Science Research Foundation of Aier Eye Hospital Group (YH).

## Conflict of Interest

The authors declare that the research was conducted in the absence of any commercial or financial relationships that could be construed as a potential conflict of interest.

## Publisher's Note

All claims expressed in this article are solely those of the authors and do not necessarily represent those of their affiliated organizations, or those of the publisher, the editors and the reviewers. Any product that may be evaluated in this article, or claim that may be made by its manufacturer, is not guaranteed or endorsed by the publisher.
