# Supplementary material for: Investigation of Correlations Between Optical Coherence Tomography Biomarkers and Visual Acuity in X-Linked Retinoschisis
Source: Front Med (Lausanne). 2022 Jan 27;8:734888. doi: 10.3389/fmed.2021.734888 (PMC8828641; doi:10.3389/fmed.2021.734888)
Supplement: Supplementary file 1 [file Image_1.pdf]

Supplementary materials for

Investigation of Correlations Between Optical Coherence Tomography Biomarkers and Visual Acuity in X-Linked Retinoschisis

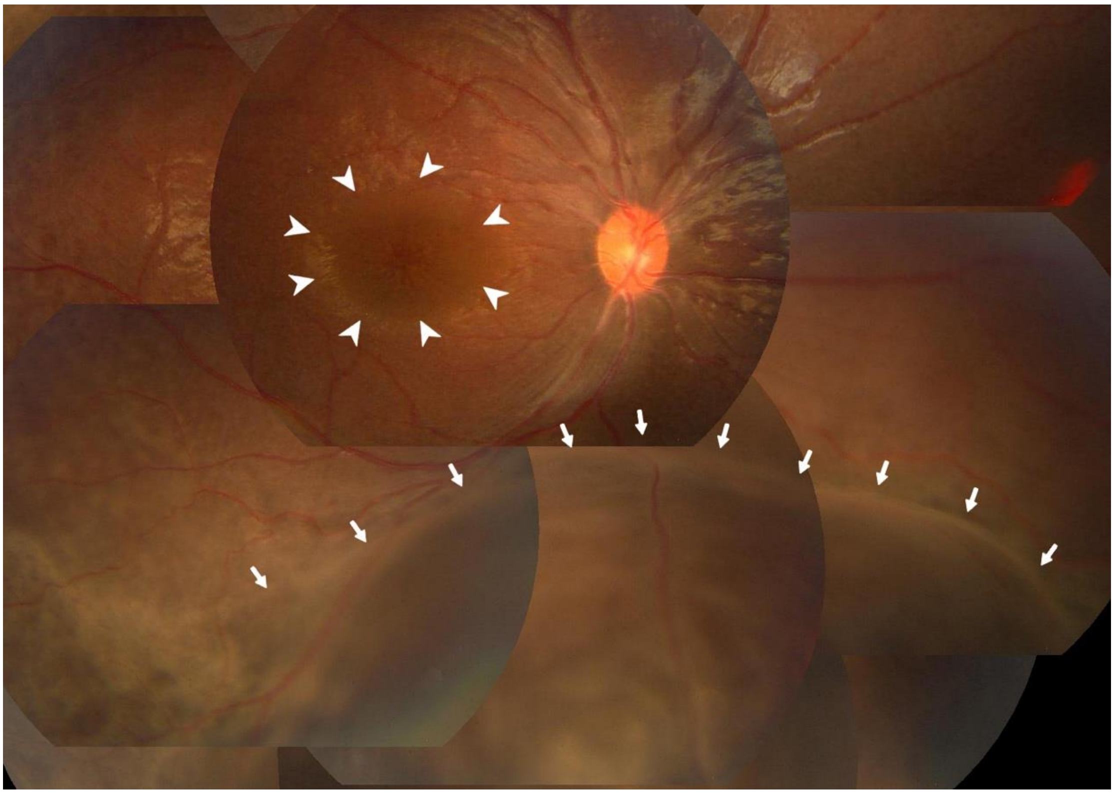

Figure S1. Fundus photograph of an XLRS patient with spoke-like macular retinoschisis (arrowheads) and peripheral retinoschisis (arrows).
